# Juvenile Recurrent Parotitis: Video-Documented Sialendoscopy

**DOI:** 10.3390/jcm12216842

**Published:** 2023-10-30

**Authors:** David Soriano-Martín, Luis García-Consuegra, Luis Junquera, Sara Reda, Sonsoles Junquera

**Affiliations:** 1 Department of Oral and Maxillofacial Surgery, Central University Hospital of Asturias, 33011 Oviedo, Spain; 2 Department of Oral and Maxillofacial Surgery, Faculty of Medicine and Health Sciences, University of Oviedo, C/. Catedrático Serrano s/n., 33006 Oviedo, Spain; 3 Department of Otolaringology, 12 de Octubre University Hospital, 28041 Madrid, Spain; 4 Department of Radiology, San Agustín University Hospital, 33401 Avilés, Spain

**Keywords:** sialendoscopy, juvenile recurrent parotitis, sialadenitis, salivary gland

## Abstract

Juvenile recurrent parotitis (JRP) is characterised by recurrent episodes of painful parotid swelling in children. JRP is the second most common cause of parotitis in childhood, behind only paramyxovirus. The prevention of recurrent attacks represents the most dramatic and serious aspect of this pathology. Since 2004, different authors have evaluated sialendoscopy for the diagnostic and therapeutic management of JRP. In this paper, we share our clinical experience of the use of sialendoscopy for the treatment of JRP. We document with video sialendoscopy the glandular pathology in four children with a mean age of 11.5 years, who had suffered from 3–6 episodes/year of inflammation prior to treatment. The use of sialendoscopy in our patients was effective in preventing recurrences. For the first time, the videosialendoscopy of a series of children diagnosed with JRP is documented in the literature.

## 1. Introduction

Juvenile recurrent parotitis (JRP) is defined as a recurrent parotid inflammation of a non-obstructive and non-suppurative nature in a child aged 1–16 years. It presents as unilateral or bilateral parotid gland inflammation with two or more episodes before puberty [1,2,3]. Excluding viral infections, salivary gland diseases are rare in children and adolescents. JRP is the second most frequent cause of parotitis in childhood, behind only paramyxovirus (mumps) [4]. The literature describes young SARS-CoV-2-positive patients whose first clinical manifestation was an acute non-suppurative episode of parotitis. It is suspected that the virus directly infects salivary gland tissues or triggers an inflammatory response that causes swelling. Supportive treatment, such as hydration and pain control, may sometimes be sufficient [5,6].

JRP is usually self-limiting, with two described peaks of incidence, between 3 and 6 years of age and between 9 and 11 years of age, and classically resolves in adolescence. It is characterised by recurrent episodes of swelling and/or pain associated with fever and malaise, and is usually unilateral [7,8].

The diagnosis of JRP is based on the clinical picture and can be confirmed through ultrasound (US). The non-invasive nature of US makes it an ideal imaging modality for children. Expected ultrasound findings for JRP include scattered hypoechoic foci (termed “Swiss cheese” or “moth-eaten”) [9].

Conservative treatment of acute episodes of JRP consists of sialagogues, warm compresses, massage and analgesics. In persistent or severe episodes, antibiotics may be administered, but the practice is controversial and not standardised [4,9]. In fact, the prevention of recurrent attacks represents the most dramatic and serious aspect of this pathology. Recurrences not only significantly influence the quality of life but can also lead to progressive destruction of the gland, and in rare cases to major interventions such as superficial or total parotidectomy. Sialendoscopy has emerged as a new treatment modality for recurrent cases of JRP to simultaneously confirm the diagnosis through direct visualisation and provide treatment through canalicular lavage with or without steroid infusion [8,9].

Traditional conservative treatments for JRP, although effective in the short term, have often resulted in recurrence of symptoms [8]. Sialendoscopy offers the possibility of minimally invasive, safe and effective treatment that reduces the proportion of patients experiencing disease recurrence [10]. Nevertheless, sialendoscopy is an option in the management of JRP that helps to confirm the diagnosis and also to intervene therapeutically. However, although there are other advantages, the definitive value of salivary gland endoscopy requires continued evaluation in new prospective studies [11].

In this article, we share the clinical experience of using sialendoscopy for treatment of JRP. The findings in five sialendoscopic procedures are documented on video. To our knowledge, this is the first series of patients with JRP documented with video sialendoscopy.

## 2. Video-Sialendoscopy: Case Series

Four children with JRP were treated with sialendoscopy, between January 2020 and December 2022, at a referral hospital with a target population of 1,005,000 (Table 1).

Criteria for the diagnosis of JRP were established according to those postulated by Garavello et al. [3]: age < 16 years, unilateral or bilateral recurrent painful parotid swelling and at least 2 episodes during the last 6 months. Exclusion criteria used were obstructive lesions, dental malocclusion, Sjögren’s syndrome and IgA deficiency. In our cases, three patients had unilateral swelling and one had bilateral swelling.

The mean age of the children in our series was 11.5 years (range: 11–13 years). All children had unilateral swelling, except for case 1 (Table 1). The number of episodes of parotid swelling per year before sialendoscopy ranged from 3 to 6. In all children, the diagnosis of JRP is based on the clinical picture and can be confirmed through ultrasonography. All ultrasound scans were performed by the same radiologist. In the current literature, there is no classification system of ultrasound findings according to the severity of JRP. Some authors [12,13] proposed a simplified scoring system for salivary glands in which parenchymal homogeneity is graded from 0 to 3 as follows: 0 (normal), 1 (mild inhomogeneity), 2 (several rounded hypoechoic lesions) and 3 (numerous or confluent hypoechoic lesions). The majority of the glands were identified through ultrasound as grade 1 or 2. Only case 3 reached a score of 3. The patient with bilateral parotid involvement was identified as grade 0 [12] on the right side (2 episodes of parotid inflammation/year) and grade 2 [12] on the left side (5 episodes/year) (Figure S1A,B).

The sialendoscopy and general anaesthesia procedure was described in detail to the patients’ parents before they signed the informed consent forms. All procedures were performed during an asymptomatic interval by the same specialist (L.G.C) with a semi-flexible 1.1 mm Karl Storz endoscope (Tuttlingen, Germany). Before starting the procedure, we must take into account the following: (1) the screen should be in a position in front of the surgeon and aligned at eye level, (2) the camera should be oriented with the endoscope and (3) the system should be flushed in order to avoid air bubbles. Only one patient underwent surgery under local anaesthesia; the rest were performed under general anaesthesia. In the case of sialendocopy performed under local anaesthesia, two pulses of lidocaine spray 10% (10 mg lidocaine/pulse) are applied to the papilla area and 1 millilitre of lidocaine 1%, without adrenaline, is infiltrated into Stensen’s duct. When the papilla is well identified, the 00 dilator is gently passed posteriorly, superiorly and laterally to the parotid (approximately 1.5 cm deep). Subsequently, the 0 and conic dilators are introduced (Marchal set). The sialendoscope is inserted and the duct should be directly visualised. For duct navigation, the surgeon should pull the cheek forwards to circumvent the masteric bend. The probes should not be forced, and ductal exploration should be performed with direct vision. The findings are explained in Table 1 and displayed in Videos S1.1, S1.2 and S2. In addition, Figures S2 and S3 show specific sialendoscopic findings of cases 3 and 4 respectively.

The irrigation solution during sialendocopy was saline solution in all cases, not exceeding 20 cc per gland in any case. In cases 2, 3 and 4, the final lavage was performed with 100 mg of hydrocortisone. When necessary, a high-pressure balloon catheter was introduced through the sialendoscope to dilate the stenosed duct. There were no intraoperative complications.

The postoperative follow-up period ranged from 2 months (case 4) to 24 months (case 1). To date, no child has had a new episode of parotid inflammation.

## 3. Discussion

Originally, JRP was attributed to congenital dilatations and malformations and/or recurrent infections [14], but a multifactorial approach to the aetiology is now more widely accepted. Genetically, JRP has an autosomal dominant pattern with incomplete penetrance and variable expression [15].

Sialendoscopy is a relatively novel and promising approach to salivary gland pathologies in which technological advances have allowed the valuable opportunity to view the interior of the ductal system. First introduced in the 1990s by Katz et al. in France and Königsberger et al. in Germany, videoendoscopy of the salivary glands became an established procedure after its standardisation and wide dissemination by Marchal and Nahlieli [8,16,17].

Recently, multiple international reports have been published describing sialendoscopy as an effective treatment for JRP [3,8,9,18]. According to the literature, paediatric ductal diameter does not differ significantly from that of adults, so direct ductal visualisation and interventional procedures can be performed at all ages. Sialendoscopy provides a minimally invasive approach for the management of JRP, allowing intraluminal endoscopic visualisation of the ductal system and a vehicle to administer irrigation and lavage within the gland under direct observation [8,10]. In our cases, the most recognised sialendoscopic finding was represented by a pale, avascular and stenotic Stensen’s duct. Canzi et al. [8] concur with our observations. In their review of the literature, the most relevant sialendoscopic finding was the white appearance of the wall and the lack of vascularisation in the ductal layer (mean 75%). Confined or diffuse strictures and multiple fibrinous debris/mucous plugs were observed in a high percentage of children (mean 56% and 45%, respectively) [8,19,20].

The literature describes sialectasia as the most frequent ultrasound finding for the diagnosis of JRP (mean 84%) [8]. The presence of pathological findings on ultrasound of the contralateral parotid gland increases the likelihood of needing additional sialendoscopy [13]. Bilateral sialendoscopy was performed in the first patient in our series. However, sialendoscopic findings on the right side showed no alterations (Video S1.1: Supplementary Data). In our study, most of the children had mild ultrasound findings. US is a well-described imaging modality for both lithiasic and non-lithiasic cases; however, there is a lack of a classification system specifically designed for JRP. Therefore, we decided to classify the ultrasound findings of our patients according to an existing scoring system for primary Sjögren’s syndrome [12].

Histologically, in patients with JRP, there are intraductal cystic dilatations of the peripheral ducts with periductal lymphocytic infiltration, termed sialectasia. The ecstatic ducts are usually 1–2 mm in diameter and have a typical whitish appearance of the ductal layer without the cover of healthy blood vessels, compared to a normal gland [21,22].

In the literature, most sialendoscopies were performed under general anaesthesia. Kanerva et al. [23] performed sialendoscopy under local anaesthesia in patients older than 10 years and Konstantinidis et al. [20] performed it in patients older than 8 years. Sedation or sedation and local anaesthesia were used in 10% of the procedures [19,24]. In patients undergoing sialendoscopy under general anaesthesia, the patient was orally or nasally intubated in the supine position.

## 4. Conclusions

The present work documents for pedagogical purposes in video format the sialendoscopies of four children with JRP. The procedure allows direct visualisation of the duct for diagnostic purposes and offers treatment by breaking the cycle of inflammation through dilatation of the ductal strictures (with hydrostatic pressure, balloons or the endoscope itself), removal of ductal debris with irrigation and instillation of anti-inflammatory drugs.

## Figures and Tables

**Table 1:** Clinical and sialendoscopic data of children (M: male. F: female. R: right site. L: left site. SS: saline solution).

Patient No.	1	2	3	4
**Age**	11	11	11	13
**Gender**	M	F	M	F
**Gland side**	R/L	R	L	L
**Recurrences/years before sialendoscopy**	5	6	5	3
**Type of anaesthesia**	Local	General	General	General
**Sialendoscopic features**	R: normal. L: mucous plug.	Mucous plug, pale duct.	Mucous plug, sialodochitis.	Duct stenosis, sialodochitis.
**Complications**	No	No	No	No
**Irrigation**	SS	SS + 100 mg hydrocortisone	SS + 100 mg hydrocortisone	SS + 100 mg hydrocortisone
**Follow-up (moths)**	24	13	5	2
**Recurrences**	No	No	No	No

## Data Availability

Not applicable.

## Supplementary Materials

The following supporting information can be downloaded at https://www.mdpi.com/article/10.3390/jcm12216842/s1. Annex Videos S1.1, S1.2 and S2 are representative videos of the pathology and Figures S1–S3 are figures with the ultrasound findings of case 1 and screen captures of the sialendoscopies in the different cases presented. Videos S1.1 and S1.2: Case 1. An 11-year-old boy with bilateral JRP. The patient was identified as grade 0 on the right side (2 episodes of parotid inflammation/year) and grade 2 on the left side (5 episodes/year) (Figure S1A,B) after sialendoscopy: (1) Video S1.1 shows an unaltered Stensen’s duct with adequate diameter and no mucoid content. (2) Video S1.2. The presence of a mucous plug in the salivary duct obstructs saliva excretion. In this patient, no stenosis was observed. During the operation, he was irrigated with saline solution, without a final corticosteroid lavage. After two years of follow-up, no recurrences were observed. Video S2: Case 2. An 11-year-old girl with six episodes of rigid parotid swelling in the year prior to surgery. The main finding identified on sialendoscopy was the presence of a pale duct and mucous plug with mild sialodochitis. The patient was irrigated with saline solution and hydrocortisone was administered at the end of the procedure (Table 1). At 13 months follow-up, no recurrences were observed. Figure S1A. Ultrasonographic findings (Case 1). (A) Right parotid gland: Heterogeneous echogenic structure with small patchy hypoechoic areas. One more marked than the other with its characteristic slightly hyperechoic parenchyma. (B) Left parotid gland in the same patient. Note the markedly hypoechoic parenchyma compared to the contralateral side. Figure S1B. Sialendoscopic view from inside Stensen’s duct (Case 1 and Videos S1.1 and S1.2: Supplementary Material). (A) Sialendoscopic findings of the right parotid gland showed no alterations. (B) Left parotid gland, in the same patient, showing mucus plug with pale mucosa. Figure S2: Case 3. An 11-year-old boy with left JRP: Inflammation of the duct wall. Figure S3: Case 4. A 13-year-old girl with left JRP: Duct stenosis.
