# Supplementary material for: Juvenile Recurrent Parotitis: Video-Documented Sialendoscopy
Source: J Clin Med. 2023 Oct 30;12(21):6842. doi: 10.3390/jcm12216842 (PMC10649241; doi:10.3390/jcm12216842)
Supplement: Supplementary file 1 [file jcm-12-06842-s001.zip › jcm-2585259-supplementary.pdf]

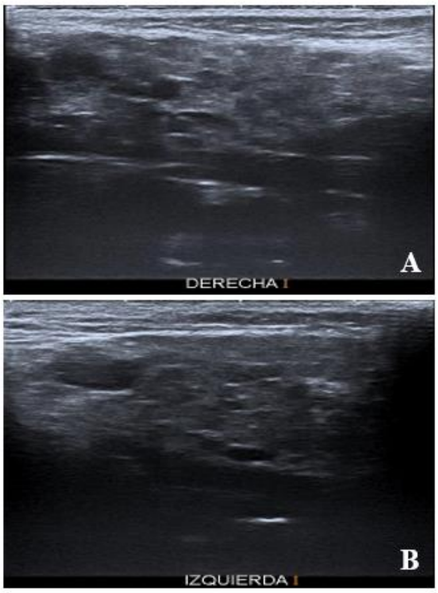

1A

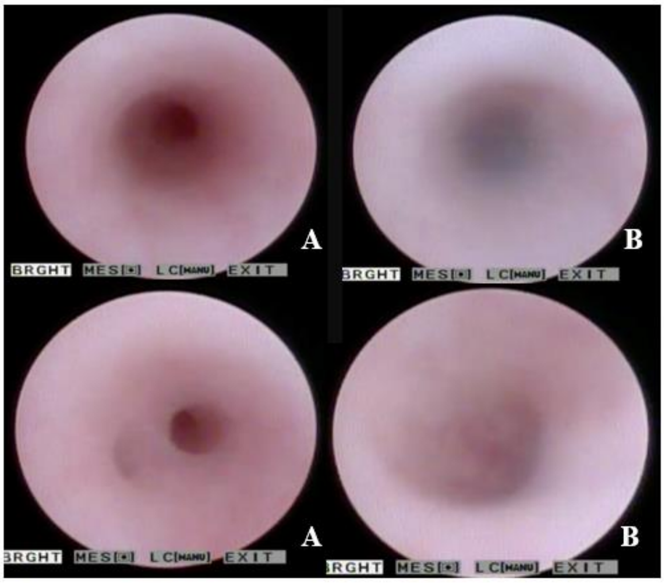

1B

Figure S1. (1A) Ultrasonographic findings (Case 1). (A) Right parotid gland: Heterogeneous echogenic structure with small patchy hypoechoic areas. One more marked than the other with its characteristic slightly hyperechoic parenchyma. (B) Left parotid gland in the same patient. Note the markedly hypoechoic parenchyma compared to the contralateral side. (**1B**) Sialendoscopic view from inside Stensen's duct (Case 1 and video S1.1 and S1.2: Supplementary material) (A) Sialendoscopic findings of the right parotid gland showed no alterations. (B) Left parotid gland, in the same patient, showing mucus plug with pale mucosa.

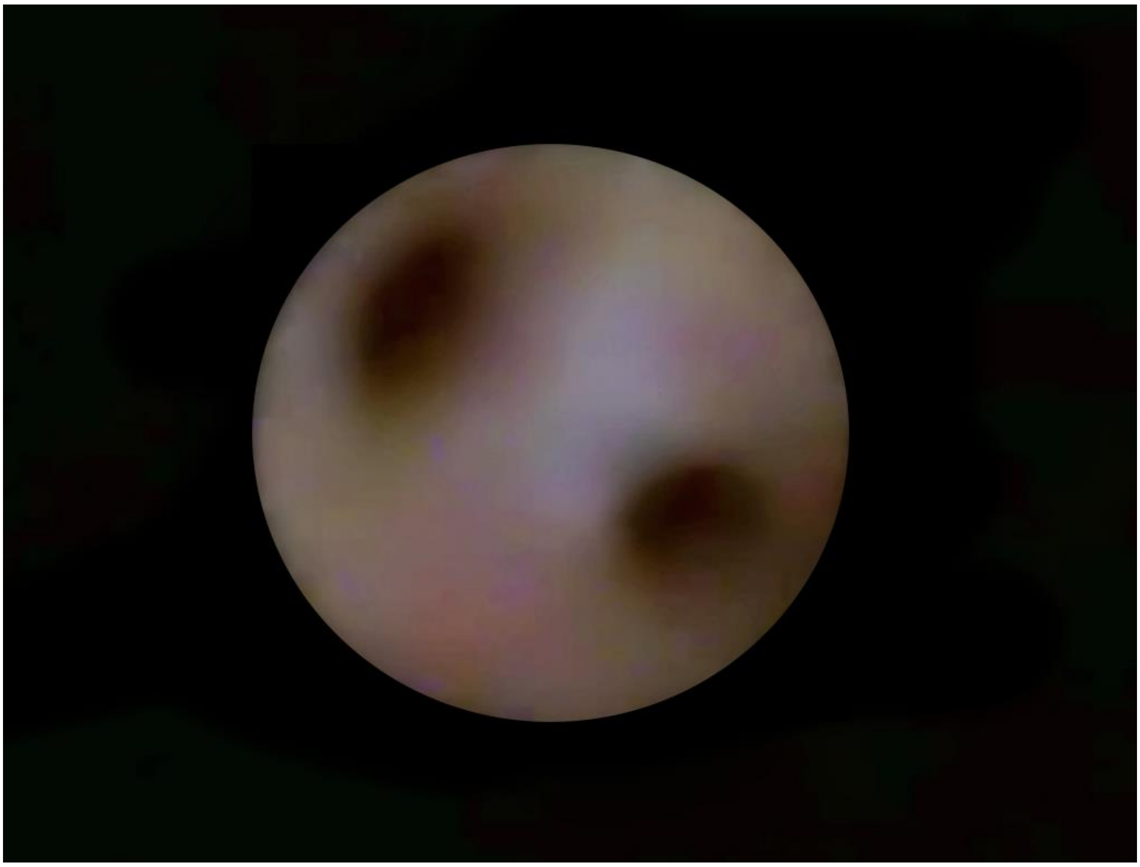

Figure S2. Case 3. 11-year-old boy with left JRP: Inflammation of the duct wall.

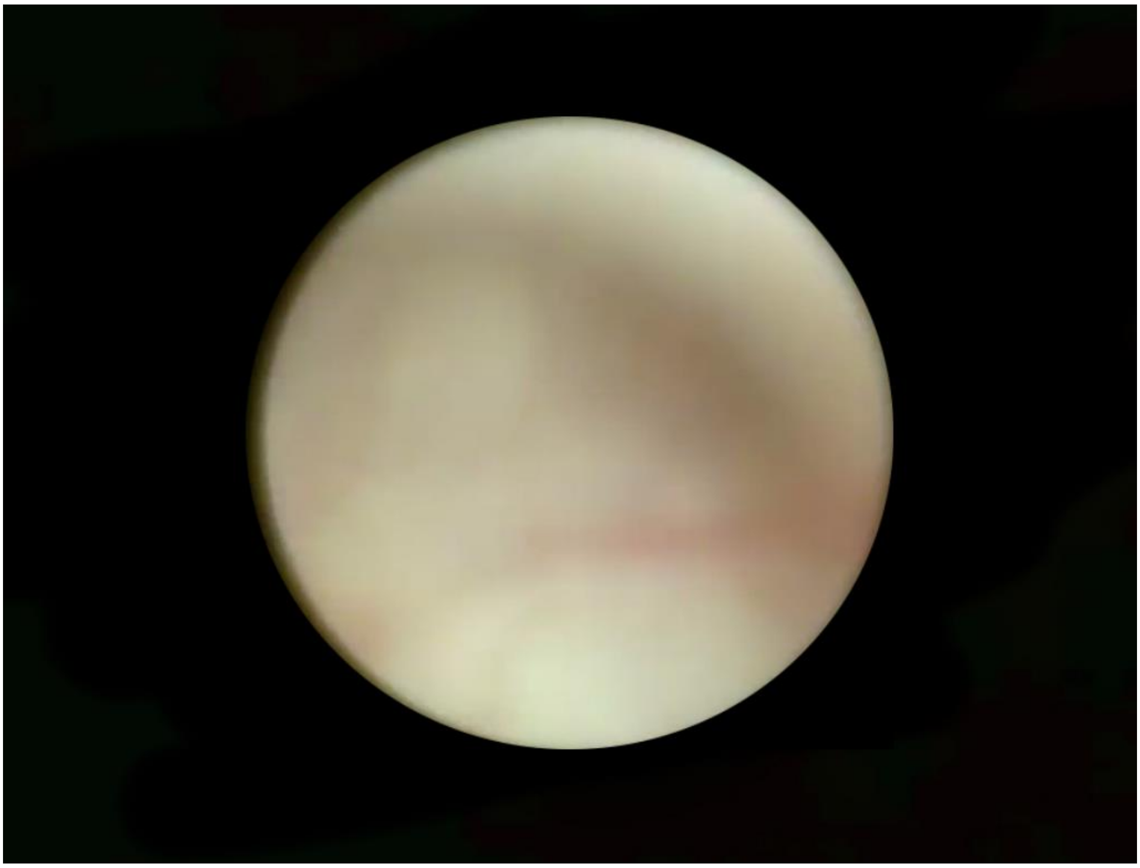

Figure S3. Case 4. 13-year-old girl with left JRP: Duct stenosis.
